# Computed Tomographic Evaluation of Cranial Suture Obliteration for Age Estimation in an Indian Population

**DOI:** 10.7759/cureus.36160

**Published:** 2023-03-14

**Authors:** Mohammed Akbar N J, Raghvendra S Shekhawat, Tanuj Kanchan, Taruna Yadav, Vikas P Meshram, Rutwik Shedge, Seshagiri Raju Vempalli, Puneet Setia

**Affiliations:** 1 Forensic Medicine and Toxicology, ESIC (Employees' State Insurance Corporation) Medical College and PGIMSR (Post Graduate Institute of Medical Sciences and Research) Bengaluru, Bengaluru, IND; 2 Forensic Medicine and Toxicology, All India Institute of Medical Sciences, Jodhpur, Jodhpur, IND; 3 Radiology, All India Institute of Medical Sciences, Jodhpur, Jodhpur, IND; 4 Forensic Medicine, School of Forensic Sciences, National Forensic Sciences University, Tirpura, Tripura, IND; 5 Forensic Medicine and Toxicology, All India Institute of Medical Sciences, Mangalagiri, Mangalagiri, IND

**Keywords:** forensic radiology, computed tomography, three stage scoring, cranial suture obliteration, age estimation, human identification, forensic anthropology

## Abstract

Background: Age estimation is a critical aspect of human identification. Age is assessed using cranial suture obliteration, pelvic morphological changes, epiphyseal fusion of long bones, dental maturation, and other standard methods.

Methods: The present study investigated three-dimensional (3D) computed tomography (CT) scans of 263 individuals (183 males and 80 females) to assess the extent of ectocranial suture closure. The assessment of obliteration was done using a three-stage scoring method. Spearman’s correlation coefficient (p < 0.05) was calculated to assess the relationship between cranial suture closure and chronological age. Simple and multiple linear regression models were developed using the cranial suture obliteration scores to estimate age.

Results: The standard errors of the estimate using multiple linear regression models developed for estimating age using obliteration scores of sagittal, coronal, and lambdoid sutures were 15.08 years in males, 13.27 years in females, and 14.74 years in the total study population.

Conclusion: This study concludes that in the absence of additional skeletal age markers, this method can be used alone or in conjunction with other well-established methods of age assessment.

## Introduction

Identification of unknown individuals is one of the primary tasks for a medicolegal professional. Estimation of age, sex, and stature constitutes the ‘Big 3’ of identification [1]. Forensic age estimation is routinely performed in cases of unidentified individuals and dismembered, mutilated, or charred human remains [2]. In the living, it is routinely performed in cases of individuals seeking asylum, armed conflict survivors, unaccompanied minors, and for assigning criminal responsibility, child labor, solicitation, and marriages [3,4].

In cases of juveniles and young adults, an epiphyseal fusion of long bones as well as dental eruption and maturation is routinely investigated to estimate age. However, with the advancement of age, these skeletal and dental indicators are of limited utility in age estimation practices. In such a scenario, age can be estimated from the epiphyseal fusion of the medial end of the clavicle, the fusion of the sternum, pubic symphyseal changes, and obliteration of cranial sutures [3,5-22].

Cranial sutures undergo obliteration in a predictable manner as the age of an individual progresses [23]. The extent of obliteration can aid medicolegal professionals in estimating the age of an individual. In 1924, Todd and Lyon studied the crania of the Hamann and Todd collection of American Whites and American Blacks to study cranial suture closure [24]. Meindl and Lovejoy (1985) graded the ectocranial suture closure on a scale of 0-3 from the Hamann and Todd collection of 236 skulls for age estimation [21]. A thorough research has been conducted on forensic age estimation using cranial sutures by Mann et al. (1991) [25], Key et al. (1994) [26], Khandare et al. (2014) [27], Singh et al. [28], Kanika et al. [29], and Mukesh et al. [30], based on the ectocranial and endocranial surface of the skull using different techniques and scoring methods. Shaikh et al. and Chandra et al. observed suture closure activity of sagittal, lambdoid, and coronal sutures for age estimation using the radiographic technique [31,32].

However, in the Indian population, the literature regarding the use of cranial suture obliteration as an indicator of age using 3D computed tomographic images of the skull is limited [27]; the extent of cranial suture obliteration for age estimation has been conducted mostly on autopsy samples using both the endocranial and ectocranial surfaces of the skull [33-35]. Computed tomography examination of cranial suture obliteration has been used in multiple studies globally, with most of the research conducted in China [12,18], Japan [11], and Germany [13].

Imaging modalities, such as computed tomography (CT), allow scientists to examine age-related changes in cranial sutures in living people. CT allows for rapid processing and faster investigation and imparts clarity in assessing these changes [6,7,22,36]. A thorough literature search indicates that there is limited research on the 3D CT-based technique of age estimation using cranial sutures in the Indian population [27]. Therefore, the goal of this work is to generate regression models by assessing cranial suture obliteration in 3D CT images for the estimation of age in an Indian population using a three-stage scoring method.

## Materials and methods

The study was conducted on patients aged 18 years and above admitted to the Department of Diagnostic and Interventional Radiology, AIIMS (All India Institute of Medical Sciences) Jodhpur, a tertiary healthcare center in India. These patients had been advised CT of the head by their treating physicians for diagnostic purposes. The patients were informed about the scope of the study in detail, and the CT images were included only after obtaining their informed consent with documented proof of age (Aadhaar card). Individuals suffering from any injuries, fractures, or developmental disorders of the skull were excluded from the study. The study population consisted of 263 participants (183 males and 80 females). The males were aged between 18 and 83 years (mean + SD: 41.22 + 17.37 years), and the females were aged between 18 and 84 years (mean + SD: 47.46 + 16.16 years). The age and sex distribution of the study population are shown in Table 1.

**Table 1:** Age and sex distribution of the study population

Age (in years)	Male	Female	Total
<20	20	3	23
21-30	47	9	56
31-40	32	21	53
41-50	27	18	45
51-60	23	9	32
61-70	26	13	39
71-80	7	5	12
>80	1	2	3
Total	183	80	263

Scanning parameters

The CT images obtained from the patients had been scanned using a DSCT-SOMATOM Definition flash 256-slice CT scanner (Siemens Medical Solutions, Erlangen, Germany). The scanning parameters were as follows: tube voltage of 80 kV, tube current of 58 mAs, and slice thickness of 0.6 mm. All the CT images obtained were coded to blind the examiner from the identities of the participants. All three cranial sutures (sagittal, coronal, and lambdoid) were studied using the Radiant Dicom software. Anatomical landmarks namely the nasion, glabella, bregma, lambda, opisthocranion, asterion, pterion, and porion were manually identified and validated on multiplanar slices along the volume-rendered (VR) pictures. The workstation software measuring tool was then used for rotation, translation, and zooming, while transparency technologies were utilized to enable exact landmark recognition. The Frankfurt Horizontal plane was designed to assist the technique, since it may be difficult to accurately position directly on the 3D surfaces. In three-dimensional CT, the Frankfurt plane functioned as the best reference plane.

Scoring of the cranial suture obliteration

The degree of cranial suture closure was assessed using a three-stage scoring system which grades cranial suture obliteration from 1 to 3. The obliteration of cranial sutures was scored as follows: 1) incipient closure indicated by the evidence of bony bridging up to 50% closure; 2) significant closure indicated by the evidence of bony bridging greater than 50%; and 3) obliteration with no trace remaining of the suture margins. The three cranial sutures, sagittal, coronal, and lambdoid, were divided into discrete parts for ease of analysis: the sagittal suture was divided into four parts, namely S1, S2, S3, and S4; the coronal suture was divided into three parts each on the right (RC1, RC2, and RC3) and left (LC1, LC2, and LC3) sides; and the lambdoid suture was divided into three parts each on the right (RL1, RL2, and RL3) and left (LL1, LL2, and LL3) sides.

## Results

Statistical analysis

All the statistical analyses were conducted using the IBM Statistical Package for Social Sciences (SPSS version 26.0). Since ordinal values (obliteration scores) were assigned to cranial suture obliteration, the data set was considered to follow non-normal distribution, and hence non-parametric tests were used for further analysis. Sex differences in cranial suture obliteration were calculated using the Mann-Whitney U test, and bilateral differences, wherever applicable, were calculated using the Wilcoxon signed-rank test. Spearman’s rho was calculated as a measure of the correlation between the fusion score of the cranial suture and the chronological age. In addition, simple and multiple linear regression models to estimate age using cranial suture obliteration scores were generated.

On the initial 50 individual CT pictures and the subsequent 50 randomly selected CT images of the participants, respectively, intra- and interobserver errors were determined using Cohen's κ. α was set to 0.005 for all the observations. Kappa values for intraobserver errors were found to be 0.92, indicating strong agreement between the principal investigator's primary and secondary observations. Additionally, it was noted that the interobserver error kappa result was 0.89, indicating strong agreement between the principal investigator and individual observer.

Statistically significant (p < 0.05) sex differences in cranial suture closure were observed in the first part of the sagittal suture (S1), the first and second parts of the right and left coronal sutures (RC1, RC2, LC1, and LC2), and the first part of the left lambdoid suture (LL1). Statistically significant bilateral differences (Z = -3.973, p < 0.001) in cranial suture obliteration were observed in the first part of the coronal suture on both sides (RC1 and LC1). As statistically significant sex and bilateral differences were observed in various parts of cranial sutures, further analysis was conducted separately for males and females, involving all the sutures.

A statistically significant correlation was observed between chronological age and cranial suture obliteration scores (p < 0.001). In males, the highest coefficient of correlation was shown by the obliteration score of sagittal suture part 2 (R = 0.512; p < 0.001), while in females, the obliteration score of sagittal suture part 1 showed the highest degree of correlation with chronological age (R = 0.499; p < 0.001).

Simple linear regression models to estimate age using obliteration scores of each of the cranial suture parts analyzed in the present study were generated for males, females, and the total study population. The least standard error of the estimate was shown by the regression model derived using the obliteration score of the left lambdoid suture part 2 in males (standard error of estimate (SEE) = 15.10 years), sagittal suture part 1 in females (SEE = 13.72 years), and sagittal suture part 2 for the entire study population (SEE = 14.93 years). These regression models are shown in Tables 2-4.

**Table 2:** Linear regression models to estimate age using cranial suture obliteration scores in males S1 = sagittal suture part 1; S2 = sagittal suture part 2; S3 = sagittal suture part 3; S4 = sagittal suture part 4; RC1 = right coronal suture part 1; RC2 = right coronal suture part 2; RC3 = right coronal suture part 3; LC1 = left coronal suture part 1; LC2 = left coronal suture part 2; LC3 = left coronal suture part 3; RL1 = right lambdoid suture part 1; RL2 = right lambdoid suture part 2; RL3 = right lambdoid suture part 3; LL1 = left lambdoid suture part 1; LL2 = left lambdoid suture part 2; LL3 = left lambdoid suture part 3; SEE = standard error of estimate.

Variable	Model	R	SEE (years)
S1	10.945 X S1 + 20.226	0.231	16.96
S2	15.345 X S2 + 16.231	0.512	15.19
S3	11.266 X S3 + 18.932	0.226	16.93
S4	13.985 X S4 + 17.223	0.442	15.71
RC1	19.529 X RC1 + 2.694	0.247	16.83
RC2	11.470 X RC2 + 18.968	0.199	17.03
RC3	8.701 X RC3 + 23.247	0.220	16.95
LC1	19.529 X RC1 + 2.694	0.247	16.83
LC2	12.062 X LC2 + 17.886	0.218	16.98
LC3	8.867 X LC3 + 22.855	0.223	16.95
RL1	14.373 X RL1 + 17.027	0.412	15.81
RL2	15.715 X RL2 + 16.658	0.469	15.33
RL3	9.097 X RL3 + 27.051	0.275	16.75
LL1	15.141 X LL1 + 15.736	0.435	15.629
LL2	16.183 X LL2 + 16.016	0.372	15.10
LL3	8.826 X LL3 + 27.376	0.269	16.81

**Table 3:** Linear regression models to estimate age using cranial suture obliteration scores in females S1 = sagittal suture part 1; S2 = sagittal suture part 2; S3 = sagittal suture part 3; S4 = sagittal suture part 4; RC1 = right coronal suture part 1; RC2 = right coronal suture part 2; RC3 = right coronal suture part 3; LC1 = left coronal suture part 1; LC2 = left coronal suture part 2; LC3 = left coronal suture part 3; RL1 = right lambdoid suture part 1; RL2 = right lambdoid suture part 2; RL3 = right lambdoid suture part 3; LL1 = left lambdoid suture part 1; LL2 = left lambdoid suture part 2; LL3 = left lambdoid suture part 3; SEE = standard error of estimate.

Variable	Model	R	SEE (years)
S1	20.976 X S1 + 3.937	0.499	13.72
S2	13.607 X S2 + 23.309	0.480	14.08
S3	10.359 X S3 + 25.838	0.288	15.56
S4	12.045 X S4 + 25.029	0.409	14.61
RC1	25.990 X RC1 – 6.467	0.442	14.11
RC2	23.456 X RC2 – 0.916	0.496	13.76
RC3	13.229 X RC3 + 18.524	0.413	14.81
LC1	25.990 X LC1 – 6.467	0.442	14.12
LC2	20.860 X LC2 + 4.699	0.456	14.10
LC3	12.971 X LC3 + 19.251	0.421	14.77
RL1	10.941 X RL1 + 27.086	0.332	15.22
RL2	12.270 X RL2 + 26.911	0.410	14.54
RL3	6.211 X RL3 + 37.370	0.179	15.95
LL1	10.941 X LL1 + 27.086	0.332	15.22
LL2	11.134 X LL2 + 28.953	0.372	14.84
LL3	6.211 X LL3 + 37.370	0.179	15.95

**Table 4:** Linear regression models to estimate age using cranial suture obliteration scores in total study population S1 = sagittal suture part 1; S2 = sagittal suture part 2; S3 = sagittal suture part 3; S4 = sagittal suture part 4; RC1 = right coronal suture part 1; RC2 = right coronal suture part 2; RC3 = right coronal suture part 3; LC1 = left coronal suture part 1; LC2 = left coronal suture part 2; LC3 = left coronal suture part 3; RL1 = right lambdoid suture part 1; RL2 = right lambdoid suture part 2; RL3 = right lambdoid suture part 3; LL1 = left lambdoid suture part 1; LL2 = left lambdoid suture part 2; LL3 = left lambdoid suture part 3; SEE = standard error of estimate.

Variable	Model	R	SEE (years)
S1	15.727 X S1 + 12.202	0.333	16.19
S2	15.155 X S2 + 17.764	0.512	14.93
S3	11.668 X S3 + 19.649	0.257	16.63
S4	13.746 X S4 + 18.919	0.439	15.46
RC1	23.822 X RC1 – 4.617	0.328	16.12
RC2	17.252 X RC2 + 9.007	0.309	16.28
RC3	10.952 X RC3 + 20.089	0.293	16.46
LC1	23.822 X LC1 – 4.617	0.328	16.12
LC2	10.599 X LC2 + 10.424	0.307	16.29
LC3	10.988 X LC3 + 20.014	0.294	16.46
RL1	13.834 X RL1 + 19.079	0.412	15.70
RL2	14.849 X RL2 + 19.405	0.460	15.22
RL3	8.512 X RL3 + 29.686	0.252	16.69
LL1	14.342 X LL1 + 18.196	0.424	15.57
LL2	14.749 X LL2 + 19.677	0.500	15.18
LL3	8.277 X LL3 + 29.994	0.244	16.72

The multiple linear regression models for the estimation of age using the obliteration scores of sagittal, coronal, and lambdoid sutures for males, females, and the total study population are shown in Table 5. On the sagittal suture, the standard error of the estimate was 15.17 years for males, 13.27 years for females, and 14.74 years for the total sample. On the right coronal suture, the standard error of the estimate was 16.56 years for males, 13.34 years for females, and 15.65 years for the total sample. On the left coronal suture, the standard error of the estimate was 16.53 years for males, 13.44 years for females, and 15.64 years for the total sample. On the right lambdoid suture, the standard error of the estimate was 15.33 years for males, 14.73 years for females, and 15.20 years for the total sample. On the left lambdoid suture, the standard error of the estimate was 15.08 years for males, 15 years for females, and 15.12 years for the total sample.

**Table 5:** Multiple linear regression models to estimate age using sagittal, coronal, and lambdoid suture obliteration scores S1 = sagittal suture part 1; S2 = sagittal suture part 2; S3 = sagittal suture part 3; S4 = sagittal suture part 4; RC1 = right coronal suture part 1; RC2 = right coronal suture part 2; RC3 = right coronal suture part 3; LC1 = left coronal suture part 1; LC2 = left coronal suture part 2; LC3 = left coronal suture part 3; RL1 = right lambdoid suture part 1; RL2 = right lambdoid suture part 2; RL3 = right lambdoid suture part 3; LL1 = left lambdoid suture part 1; LL2 = left lambdoid suture part 2; LL3 = left lambdoid suture part 3; SEE = standard error of estimate.

Suture	Sex	Model	SEE (years)
Sagittal	Male	1.467 X S1 + 11.366 X S2 – 1.072X S3 + 5.670 X S4 + 12.288	15.17
	Female	15.451 X S1 + 9.921 X S2 – 3.798 X S3 – 0.272 X S4 + 6.229	13.27
	Total population	6.049 X S1 + 10.641 X S2 – 1.856 X S3 + 4.808 X S4 + 8.693	14.74
Coronal	Male	Right coronal: 15.506 X RC1 + 4.181 X RC2 + 6.878 X RC3 – 11.686	16.56
		Left coronal: 14.929 X LC1 + 5.316 X LC2 + 6.766 X LC3 – 12.528	16.53
	Female	Right coronal:- 13.329 X RC1 + 12.723 X RC2 + 4.929 X RC3 – 17.217	13.34
		Left coronal: 14.968 X LC1 + 9.498 X LC2 + 5.875 X LC3 – 15.844	13.44
	Total population	Right coronal: 16.079 X RC1 + 7.129 X RC2 + 6.714 X RC3 – 17.313	15.65
		Left coronal: 16.121 X LC1 + 7.038 X LC2 + 6.743 X LC3 – 17.226	15.64
Lambdoid	Male	Right lambdoid: 4.843 X RL1 + 12.235 X RL2 – 0.075RL3 + 14.063	15.33
		Left lambdoid: 5.078 X LL1 + 13.091 X LL2 – 0.975 X LL3 + 13.813	15.08
	Female	Right lambdoid: 0.947 X RL1 + 11.852 X RL2 – 0.529 X RL3 + 26.706	14.73
		Left lambdoid: 3.330 X LL1 + 8.746 X LL2 + 0.524 X LL3 + 25.870	15.00
	Total population	Right lambdoid: 4.534 X RL1 + 11.595 X RL2 + 0.032 X RL3 + 16.673	15.20
		Left lambdoid: 5.562 X LL1 + 10.924 X LL2 – 0.131 X LL3 + 16.298	15.12

**Figure 1:**
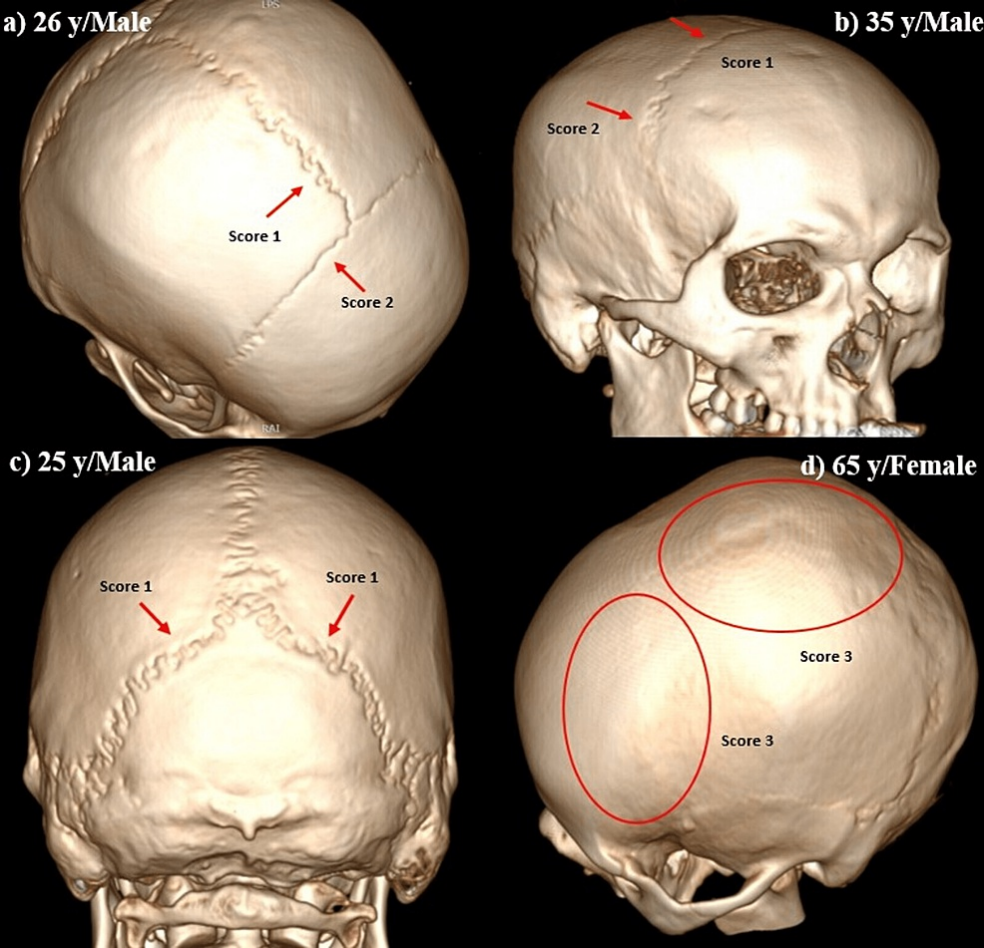
Three-dimensional CT image of skull indicating suture closure a) Score 1 of the sagittal suture and score 2 of the coronal suture, b) scores 1 and 2 in the coronal suture on right side, c) score 1 on the lambdoid suture, and d) score 3 on the coronal and sagittal suture. The red arrows indicate the cranial suture closure of scores 1 and 2, and the red circles indicate the complete closure of the suture with a score of 3.

**Figure 2:**
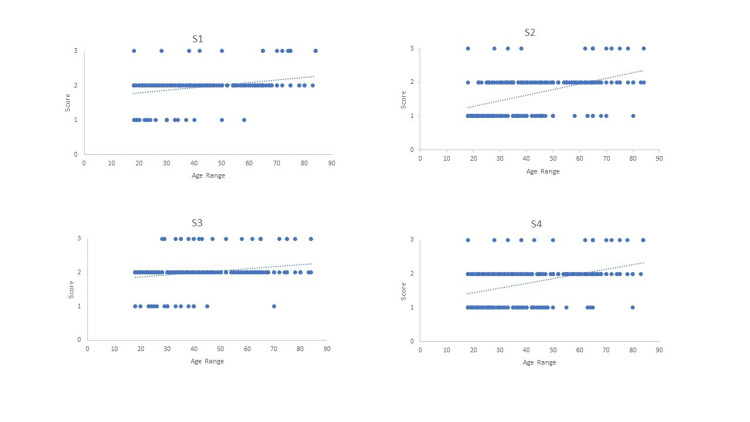
Scatter plot showing linear regression models of age estimation using sagittal suture S1 = sagittal suture part 1; S2 = sagittal suture part 2; S3 = sagittal suture part 3; S4 = sagittal suture part 4.

**Figure 3:**
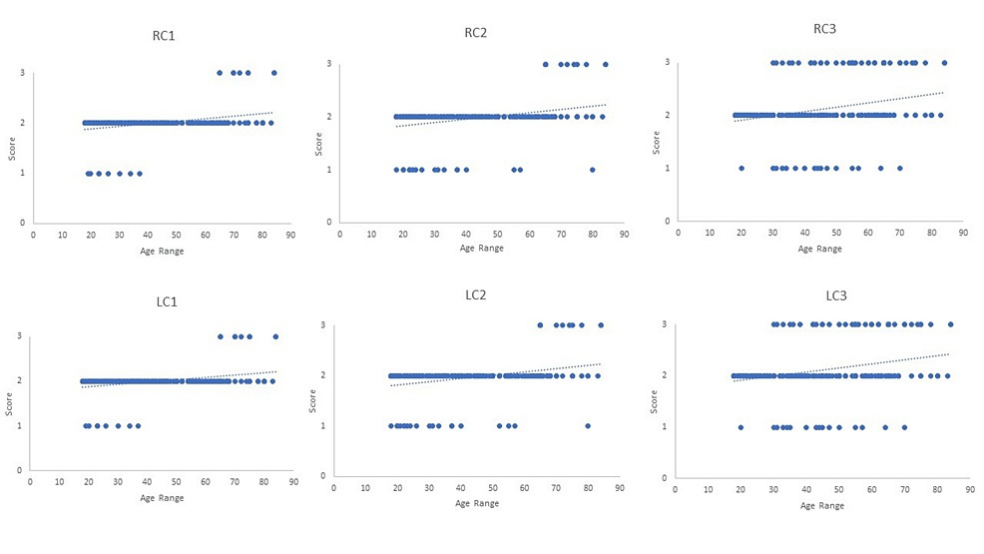
Scatter plot showing linear regression models of age estimation using coronal suture RC1 = right coronal suture part 1; RC2 = right coronal suture part 2; RC3 = right coronal suture part 3; LC1 = left coronal suture part 1; LC2 = left coronal suture part 2; LC3 = left coronal suture part 3.

**Figure 4:**
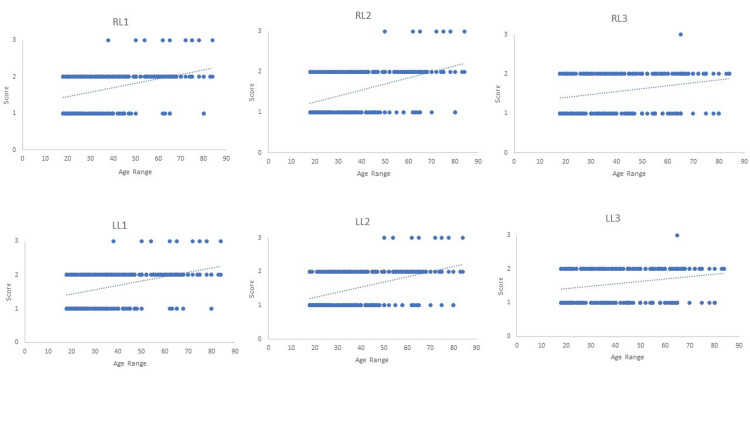
Scatter plot showing linear regression models of age estimation using lambdoid suture RL1 = right lambdoid suture part 1; RL2 = right lambdoid suture part 2; RL3 = right lambdoid suture part 3; LL1 = left lambdoid suture part 1; LL2 = left lambdoid suture part 2; LL3 = left lambdoid suture part 3.

Three-dimensional representations of different stages of cranial suture obliteration are shown in Figure 1. A scatter plot showing the linear regression models of age estimation using sagittal, coronal, and lambdoid sutures is shown in Figures 2-4.

## Discussion

Adult age and sex estimates are crucial in forensic practice. The Acsádi and Nemeskéri method of scoring the sutures was used on the endocranial and ectocranial aspects of the skull in most of the previous examinations conducted by the majority of the researchers [19,34,35,37,38]. Most commonly used skeletal and dental indicators attain complete maturity by the time an individual reaches 30 years of age [39]; with increasing age, the accuracy in estimating a person’s age decreases [40]. While obliteration of the cranial sutures has been studied for age estimation for over a century, many experts believe it to be an unreliable predictor of age [17]. Meindl and Lovejoy suggested that both ectocranial and endocranial suture closures should be used with caution and as an adjunct to other age markers [21]. In both archaeological and forensic contexts, the cranium is most often the best-preserved portion of the skeleton [41]. Post-mortem CT (PMCT) has now been established and is widely utilized in the field of forensic anthropology in numerous research projects, including the biological anthropology of ancient and modern skeletal remains [42,43]. PMCT has been used to examine the morphology, measurements, pathologies, and damage to the skull [44,45]. In the present study, an attempt was made to implement a three-stage scoring system on the ectocranial surface of the skull using the 3D CT technique. A statistically significant correlation was found between age and suture obliteration in males, females, and the total population.

In 2009, Harth et al. [13] conducted a study to estimate age using flat-panel CT by observing the obliteration of the cranial sutures. A total of 221 calvariae were examined, and the cranial suture was divided into 14 segments. A seven-stage grading method was used for all the sutures on both sides. The inaccuracy was 30.5 years in males and 29.2 years in females; this was considerably higher as compared to the present study, which may be due to the bigger sample size in the study by Harth et al. [13]. They also stated that the flat-panel CT does not offer an advantage over other methods in age estimation due to the higher range of inaccuracy of the total population of about 31.1 years and that it could be used in conjunction with other methods.

In 2013, Chiba et al. [11] conducted a study on 125 CT images of the sagittal suture for the estimation of age. The sagittal suture was divided into four segments, and a seven-stage scoring method was used. The inaccuracy was higher than the SEE of the linear regression models developed in the present study. The study found that the degree of sagittal suture closure on cross-sectional multidetector computed tomography (MDCT) images is positively linked with age and that MDCT might be a helpful technique for estimating age, particularly in adult women, because the error was considerably lower in women.

In 2012, Wolff et al. [46] investigated the accuracy of three methods for estimating skeletal age in the Hungarian population. On the ectocranial suture, Meindl and Lovejoy's approach was used [21], while on the endocranial suture, they applied Acsádi and Nemeskéri’s method [37]. The ectocranial suture closure error varied from 4.35 to 45.57 years, while the endocranial suture closure error varied from 8.32 to 23.63 years. The age group of 31-40 years had the lowest rate of inaccuracy; this demonstrates that cranial suture closure occurs most frequently between the ages of 30 and 40 and that endocranial suture closure is more reliable than ectocranial closure.

In 2018, Ruengdit et al. [19] investigated suture closure procedures on 175 Thai dry crania. The crania were subjected to three approaches for estimating age using the cranial suture: Meindl and Lovejoy (1985) [21], Mann et al. (1991) [25], and Acsádi and Nemeskéri (1970) [37]. The inaccuracy of the Meindl and Lovejoy method was 21.2 years in males, 22.1 years in females, and 21.6 years in the total population; the inaccuracy of the Acsádi and Nemeskéri approach was 13.3 years in males, 14.8 years in females, and 14 years in the total population; and finally, the inaccuracy of the Mann method was 13.8 years, 19.7 years in females, and 16.5 years in the total population. Therefore, the Acsádi and Nemeskéri method was superior to the other two. A three-stage scoring method was used in the present study over the ectocranial surface on the CT image of the skull. The smaller sample size and visualization of the suture in 3D images, which may allow for better visualization, may account for the lower error in this study.

In 2020, Fan et al. [12] conducted a study on the Chinese male population to estimate age using CT scans of the skull. It included 230 men whose ages ranged from 23.33 to 76.93 years old, with a mean age of 47.48 years and a standard deviation (SD) of 10.49 years. Fan et al. [12] used a seven-stage grading system for the cranial suture closure that was developed by Chiba et al. [11]. Using linear regression models, the study's accuracy was approximately 13.09 years. When compared to 13.27 years, the accuracy of their study is slightly better than that of the present one. This may be attributed to population differences and the fact that the study population of the Fan et al. [12] consisted of only males, with a sample size of 230 compared to our study of 182 male individuals.

In 2020, Qiu et al. [18] conducted a study on 220 Han adults (110 males and 110 females) by using thin-layer CT for age estimation. The cranial sutures were divided equally into two segments, and each segment was divided into 10 layers. Multiplanar reformation (MPR) images were used, and the cranial suture closure was graded on a scale of 1 to 7. The mean absolute error was 6.39 years in males, 6.16 years in females, and 6.29 years in the total population. The least standard error of estimate is due to the equal distribution of sample size between the male and female populations when compared to our study.

A comparison of the study characteristics of the available literature and the present study is shown in Table 6. Despite discrepancies in the methodologies, there appears to be a consistent pattern of increasing mean age of suture closure as age progresses. Suture closure is governed by the osteoblast-osteoclast mechanism, while bone metabolism is regulated by ossification [17].

**Table 6:** Comparison between the different studies and their standard error of estimate SEE = standard error of estimate.

Studies	Sample size			SEE (in years)		
	Male	Female	Total	Male	Female	Total
Harth et al. [13]	148	73	221	+30.50	+29.20	+31.10
Chiba et al. [11]	65	61	126	+33.55	+29.56	+31.42
Qiu et al. [18]	110	110	220	+6.39	+6.16	+6.29
Fan et al. [12]	230	-	230	+6.22	-	-
Ruengdit et al. [19]	161	85	76	+13.30	+ 14.80	+ 14.00
Present study	183	80	263	+15.08	+13.27	+14.74

CT examination of the skull for age estimate is arguably more successful than traditional methods, as it is extremely effective and less time-consuming and does not require the removal of soft tissues, which destroys the anatomy of the body. The 3D CT provided outstanding visibility and clarity of the suture closure in our research, although it did have some limitations. The scoring of the cranial suture used in the present study is simple, with only three obliteration stages/scores compared to the six or seven stages of other age estimation methods employing cranial suture obliteration. CT technology has its own set of disadvantages, such as cost, the necessity for extensive training, and the frequent risk of radiation exposure and contrast dye injection into living individuals. The CT slice thickness is also crucial in properly estimating an individual's age. The precision of the visualization decreases as the slice thickness increases, resulting in a drop in accuracy. Because the radiation dosage is one-fifth that of a normal dose CT, low-dose CT is a considerable improvement over standard dose CT. To the best of our knowledge, the regression models developed in this study were the first of their kind in the region of Northwest India.

## Conclusions

Cranial sutures undergo obliteration as age advances, which can be assessed using the three-stage scoring method. Obliteration of the cranial sutures shows a statistically significant correlation with chronological age, and it is a better predictor of age in females than in males. Linear regression models based on cranial suture obliteration stages can help forensic specialists estimate age. Because the regression models created utilizing obliteration scores of cranial sutures in this work have large standard estimates of error, these models should be utilized with caution in conjunction with other well-established techniques of age estimation during forensic identification. MDCT has the potential to be a valuable technique for establishing the age of an individual by visualizing the cranial suture closure. Studies should be conducted in different populations due to demographic differences, varied socioeconomic statuses, and other factors that influence skeletal maturity, such as diet, genetics, and environment.

## References

[1] Krogman WM, Iscan MY (1986). The Human Skeleton in Forensic Medicine, 2nd Edition. https://www.scirp.org/(S(351jmbntvnsjt1aadkposzje))/reference/ReferencesPapers.aspx?ReferenceID=1526382.

[2] Kanchan T, Krishan K (2013). Personal identification in forensic examinations. Anthropology.

[3] Schmeling A, Dettmeyer R, Rudolf E, Vieth V, Geserick G (2016). Forensic age estimation: methods, certainty, and the law. Dtsch Arztebl Int.

[4] Schmeling A, Reisinger W, Geserick G, Olze A (2006). Age estimation of unaccompanied minors. Part I. General considerations. Forensic Sci Int.

[5] Monum T, Makino Y, Prasitwattanaseree S (2020). Age estimation from ossification of sternum and true ribs using 3D post-mortem CT images in a Japanese population. Leg Med (Tokyo).

[6] Shedge R, Kanchan T, Garg PK, Dixit SG, Warrier V, Krishan K (2021). Age estimation from sternebral fusion in an Indian population: a computed tomographic evaluation. Leg Med (Tokyo).

[7] Shedge R, Kanchan T, Garg PK, Dixit SG, Warrier V, Khera P, Krishan K (2020). Computed tomographic analysis of medial clavicular epiphyseal fusion for age estimation in Indian population. Leg Med (Tokyo).

[8] Primeau C, Friis L, Sejrsen B, Lynnerup N (2012). A method for estimating age of Danish medieval sub-adults based on long bone length. Anthropol Anz.

[9] Kanchan T, Chugh V, Chugh A, Meshram V, Shedge R, Patnana AK, Krishan K (2021). Age estimation using third molar maturation based on Demirjian's criteria. Leg Med (Tokyo).

[10] Oldrini G, Harter V, Witte Y, Martrille L, Blum A (2016). Age estimation in living adults using 3D volume rendered CT images of the sternal plastron and lower chest. J Forensic Sci.

[11] Chiba F, Makino Y, Motomura A (2013). Age estimation by multidetector CT images of the sagittal suture. Int J Legal Med.

[12] Fan F, Tu M, Li R (2020). Age estimation by multidetector computed tomography of cranial sutures in Chinese male adults. Am J Phys Anthropol.

[13] Harth S, Obert M, Ramsthaler F, Reuß C, Traupe H, Verhoff MA (2009). Estimating age by assessing the ossification degree of cranial sutures with the aid of flat-panel-CT. Leg Med (Tokyo).

[14] Hisham S, Abdullah N, Mohamad Noor MH, Franklin D (2019). Quantification of pubic symphysis metamorphosis based on the analysis of clinical MDCT scans in a contemporary Malaysian population. J Forensic Sci.

[15] Schulz R, Mühler M, Mutze S, Schmidt S, Reisinger W, Schmeling A (2005). Studies on the time frame for ossification of the medial epiphysis of the clavicle as revealed by CT scans. Int J Legal Med.

[16] Kellinghaus M, Schulz R, Vieth V, Schmidt S, Schmeling A (2010). Forensic age estimation in living subjects based on the ossification status of the medial clavicular epiphysis as revealed by thin-slice multidetector computed tomography. Int J Legal Med.

[17] Ruengdit S, Troy Case D, Mahakkanukrauh P (2020). Cranial suture closure as an age indicator: a review. Forensic Sci Int.

[18] Qiu SW, Tu M, Fan F, Zhan MJ, Dong XA, Zhang K, Deng ZH (2020). Age estimation in Han adults by thin-layer CT scan of cranial sutures. Fa Yi Xue Za Zhi.

[19] Ruengdit S, Prasitwattanaseree S, Mekjaidee K, Sinthubua A, Mahakkanukrauh P (2018). Age estimation approaches using cranial suture closure: a validation study on a Thai population. J Forensic Leg Med.

[20] Kanchan T, Krishan K, Kumar GP (2013). Squamous suture: a rare case of asymmetrical closure with review of literature. Forensic Sci Int.

[21] Meindl RS, Lovejoy CO (1985). Ectocranial suture closure: a revised method for the determination of skeletal age at death based on the lateral-anterior sutures. Am J Phys Anthropol.

[22] Wink AE (2014). Pubic symphyseal age estimation from three-dimensional reconstructions of pelvic CT scans of live individuals. J Forensic Sci.

[23] Grova M, Lo DD, Montoro D, Hyun JS, Chung MT, Wan DC, Longaker MT (2012). Models of cranial suture biology. J Craniofac Surg.

[24] Todd TW, Lyon Jr DW (1925). Cranial suture closure. Its progress and age relationship. Part II: Ectocranial closure in adult males of white stock. Am J Phys Anthropol.

[25] Mann RW, Jantz RL, Bass WM, Willey PS (1991). Maxillary suture obliteration: a visual method for estimating skeletal age. J Forensic Sci.

[26] Key CA, Aiello LC, Molleson T (1994). Cranial suture closure and its implications for age estimation. Int J Osteoarchaeol.

[27] Khandare SV, Bhise SS, Shinde AB (2014). Age estimation from cranial sutures: CT scan study. Indian J Basic Appl Med Res.

[28] Singh P, Oberoi SS, Gorea R, Kapila AK (2004). Age estimation in old individuals by CT scan of skull. J Indian Acad Forensic Med.

[29] Kanika K, Aggarwal OP, Amit M (2019). Age estimation of individuals beyond 45 years of age by CT scan of skull. J Punjab Acad Forensic Med Toxicol.

[30] Mukesh G, Singh P, Rajesh V, Asawa S, Kochar SR (2011). Comparison of lambdoid suture closure by X-ray and CT scan in two states of India. J Indian Acad Forensic Med.

[31] Shaikh I, Kumar S, Gaur GP, Vyas PC (2017). Determination of age by studying radiological fusion of cranial sutures and sternum in living persons between the fourth and seventh decade. J Med Sci Clin Res.

[32] Chandra S, Dwivedy S, Sah K, Sinha S (2015). Application of modified reverse panoramic radiograph on lambdoid suture for age estimation. Quant Imaging Med Surg.

[33] Khandare SV, Bhise SS, Shinde AB (2015). Age estimation from cranial sutures: a postmortem study. Int J Healthc Biomed Res.

[34] Shetty U (2009). Macroscopic study of cranial suture closure at autopsy for estimation of age. Anil Aggrawal's Internet. J Forensic Med Toxicol.

[35] Ramanan G, Ranganathan S, Ranganathan S (2016). Determination of age by study of closure of endocranial sutures. J Evol Med Dent Sci.

[36] Warrier V, Kanchan T, Shedge R, Krishan K, Singh S (2021). Computed tomographic age estimation from the pubic symphysis using the Suchey-Brooks method: a systematic review and meta-analysis. Forensic Sci Int.

[37] Acsádi G, Nemeskéri J (1974). History of Human Life Span and Mortality. Budapest: Akadémiai Kiadó.

[38] Meyer A, van der Merwe AE, Steyn M (2021). An evaluation of the Acsádi and Nemeskéri Complex Method of adult age estimation in a modern South African skeletal sample. Forensic Sci Int.

[39] Litsas G, Lucchese A (2016). Dental and chronological ages as determinants of peak growth period and its relationship with dental calcification stages. Open Dent J.

[40] Kullman L (1995). Accuracy of two dental and one skeletal age estimation method in Swedish adolescents. Forensic Sci Int.

[41] Boldsen JL, Milner GR, Konigsberg LW, Wood JW, Hoppa RD, Vaupel JW (2022). Transition analysis: a new method for estimating age from skeletons. Paleodemography: Age Distributions from Skeletal Samples. Cambridge Studies in Biological and Evolutionary Anthropology.

[42] Brough AL, Morgan B, Robinson C, Black S, Cunningham C, Adams C, Rutty GN (2014). A minimum data set approach to post-mortem computed tomography reporting for anthropological biological profiling. Forensic Sci Med Pathol.

[43] Sakuma A, Ishii M, Yamamoto S (2010). Application of postmortem 3D-CT facial reconstruction for personal identification. J Forensic Sci.

[44] Appleby J, Rutty GN, Hainsworth SV (2015). Perimortem trauma in King Richard III: a skeletal analysis. Lancet.

[45] Cesarani F, Martina MC, Ferraris A (2003). Whole-body three-dimensional multidetector CT of 13 Egyptian human mummies. AJR Am J Roentgenol.

[46] Wolff K, Vas Z, Sótonyi P, Magyar LG (2012). Skeletal age estimation in Hungarian population of known age and sex. Forensic Sci Int.

